# Parental perceptions of participation in young adult‐focused eating disorder treatment

**DOI:** 10.1002/erv.3084

**Published:** 2024-03-21

**Authors:** Stephanie Knatz Peck, Terra Towne, Christina Wierenga, Taylor Perry, McKenzie Miller, Jessie Kim, Walter Kaye

**Affiliations:** ^1^ Eating Disorder Treatment & Research Center Department of Psychiatry University of California La Jolla San Diego USA

**Keywords:** clinical, development, family therapy, treatment

## Abstract

**Background:**

Eating disorders (ED) are serious mental illnesses affecting young adults (YA). Parent‐supported treatment for this age cohort is an important consideration given the unique developmental needs and norms of familial social support, but more research is needed to understand parental perceptions of treatment involvement.

**Methods:**

33 parent‐supports of YA with ED completed self‐report assessments at admission and discharge of participation in brief, intensive, young‐adult focused eating disorder treatment. Assessments measured programme satisfaction, parental self‐efficacy, and parent and YA report of eating disorder‐related psychopathology. Repeated measures ANOVAs were used to examine pre‐post outcome differences and between group differences among parent‐supports and their YA (i.e., the patients) on eating disorder psychopathology, clinical impairment, and family functioning using the EDEQ/P‐EDEQ Global, P‐CIA/CIA, and Family Assessment Device Family Functioning scales. Group differences across time points were examined with paired sample *t*‐tests adjusted for multiple comparisons. Changes in parental self‐efficacy were examined separately using two‐tailed paired sample *t*‐tests.

**Results:**

Parents reported high acceptability and learning, improvements in self‐efficacy, and significant reductions of YA psychopathology at post‐treatment. Parents reported comparable reductions in ED psychopathology post‐treatment, but significantly greater reductions in clinical impairment compared to YA. Measures of family functioning did not improve for either parent or YA at post‐treatment.

**Conclusion:**

Results from this study suggest that parental involvement in a YA programme is feasible and acceptable from a parental perspective and improves parental self‐efficacy.

## Highlights

- Parents of young adults (YA) with Eating disorders (ED) reported high acceptability and learning after participation in a young‐adult focused eating disorder treatment programme.
- Parents also reported improvements in self‐efficacy and reductions in their young adult's eating disorder psychopathology.
- Parent and young adult reports of reductions in eating disorder psychopathology were comparable, however parents reported greater improvements in clinical impairment compared to young adult reports.

## 1 INTRODUCTION

Eating disorders are dangerous and deadly behavioural illnesses with risky and costly medical and psychological sequelae (Arcelus et al., 2011; Demmler et al., 2020; Smink et al., 2012; Streatfeild et al., 2021). Young adulthood is a particularly vulnerable and sensitive time for ED development and maintenance, with onset most commonly occurring between the ages of 17–22 (Hoek, 2016; Solmi et al., 2022).

Young adulthood (approximately 18–25 years) has been recognized as a cohort with unique and distinctive developmental tasks and milestones (Arnett, 2000; Arnett et al., 2014). This life stage continues to be characterised by significant familial interdependence, with YAs often receiving economic, social and health‐related assistance from parental figures (Barroso et al., 2019; Wightman et al., 2013).

There is a strong developmental rationale for involving parents in YA treatment. Parental involvement is relatively standard in youth health services in accordance with published guidelines, whereas the majority of adults (≥18 years) with EDs receive individually‐oriented care (NationalGuidelineAlliance, 2017) due to legal consent processes and socio‐cultural norms surrounding autonomy. However, YAs represent a developmental “in‐between” where they are emerging *into* adulthood, and which may necessitate treatment adaptations (Potterton et al., 2019) including continued social support scaffolding from parental figures and family (Arnett, 2000). Indeed, young people cite social support as a notable aid to engaging in help‐seeking. They often fail to engage with services due to poor mental health literacy and difficulties recognising the need for help, amongst other barriers (Gulliver et al., 2010), suggesting a greater need for social support involvement.

Involving parents may facilitate treatment engagement and prevent chronicity, and reduce caregiver distress and burnout (Anastasiadou et al., 2014; van Hoeken & Hoek, 2020). Developmentally adapted models of ED care for YA, including those with elements of parent involvement have shown promise and may improve outcomes (Allen et al., 2020; Dimitropoulos et al., 2015). However, more studies are needed to evaluate parental perceptions to further assess feasibility of family‐involved care. Since parents often continue to be significant stakeholders in their YA's lives, it is important to understand their experience and perceptions of improvement for self and their YA child.

In this manuscript, we report on parent acceptability data and parent‐reported outcomes of pre‐ post‐treatment changes in ED psychopathology and parental self‐efficacy using data from parents who participated in a novel 5‐day, intensive treatment designed for YA and their parents (Young Adult Temperament Based Therapy with Support; YA‐TBT‐S) (Knatz Peck et al., 2021). Parental outcomes are compared to YA outcomes. We then conduct an exploratory analysis evaluating associations between change in parent self‐efficacy and treatment outcome.

## 2 METHODS

### 2.1 Participants and procedure

Patients who sought out the programme were self‐referred, parent‐referred, or community‐referred and consented to treatment. Criteria for admission to the programme included a primary ED diagnosis, attendance with at least one designated primary parent support, and medical stability assessed by a programme physician.

Forty‐three parent supports participated in the University of California San Diego (UCSD), Young Adult Temperament Based Therapy with Support Programme (YA‐TBT‐S) alongside their young adult child with an ED between 2017 and 2021 (39 attended in person and 4 attended virtually). Forty‐two of 44 patients completed; one patient dropped out due to her parent‐support being unable to attend, and another due to reluctance to attend with parents. This manuscript reports on data from 33 parent supports who completed both pre‐ and post‐treatment questionnaires. Parent‐ support and YA sample characteristics are summarised in Table 1, including YA sample data previously described elsewhere (Knatz Peck et al., 2021).

**TABLE 1:** Parent & patient demographics.

Parent demographics	*N (%)*	Patient demographics	*N(%)/M(SD)*
*Role of Support Completing Questionnaires*		*Gender*	
Mother	31 (93.94%)	Female	30 (90.91%)
Father	2 (6.06%)	Male	3 (9.09%)
*Support Attendance*		Age in years	19.30 (2.11)
Attended as sole support	15 (45.45%)	Duration of illness in years	4.39 (1.78)
Attended with partner or co‐parent	18 (54.55%)	Living with parents	25 (75%)
*Race*		*Eating Disorder Diagnosis*	
Caucasian	29 (87.88%)	Anorexia nervosa‐ restricting type	13 (39.39%)
Asian	2 (6.06%)	Anorexia nervosa‐ binge purge type	7 (21.21%)
Hispanic	1 (3.03%)	Avoidant restrictive food intake disorder	5 (15.15%)
Mixed race	1 (3.03%)	Bulimia nervosa	6 (18.18%)
		Other specified eating disorder	2 (6.06%)
*Marital Status*			
Married	29 (87.88%)		
Divorced	2 (6.06%)		
Separated	1 (3.03%)		
*Education*			
Bachelor's or graduate degree	26 (78.79%)		
Associate degree	3 (9.09%)		
Some college	3 (9.09%)		
High school graduate	1 (3.03%)		
*Employment Status*			
Full time employee	16 (48.48%)		
Part time employee	3 (9.09%)		
Stay at home parent/did not work	12 (36.36%)		
Retired	2 (6.06%)		
*Annual Household Income*			
Under $100,000	5 (15.15%)		
$100,001‐$150,000	7 (21.21%)		
$150,001‐$200,000	5 (15.15%)		
$200,001‐$250,000	5 (15.15%)		
Over $250,000	9 (27.27%)		
Did not specify income	2 (6.06%)		

### 2.2 Outcome measures

Parent supports and YA patients completed an online self‐report questionnaire at admission and discharge (pre‐treatment and post‐treatment). Parent assessments included the parent‐report version of the Eating Disorder Examination Questionnaire (PEDE‐Q) (Drury et al., 2023), the Clinical Impairment Assessment (CIA) (Bohn & Fairburn, 2008) adapted for parent‐report and a 7 day timeframe, the Parent Versus Anorexia Scale (PVAS) (Rhodes et al., 2005), and the McMaster Family Assessment Device (FAD) (Epstein et al., 2007). The PEDE‐Q has shown good psychometric properties in youth but has not been validated with YA. The scale was adapted to assess for eating pathology over the past 7 days due to the short‐term nature of the programme. Parent‐supports also completed the Client Satisfaction Questionnaire (CSQ) (Attkisson & Zwick, 1982) and acceptability and treatment feedback measures designed for this study (YA‐TBT‐S Acceptability) (Knatz Peck et al., 2021) at post‐treatment. The TBT‐S Acceptability measure consists of 18 items assessing degree of satisfaction with the overall programme and specific components of the treatment rated on a 5‐point Likert scale. Acceptability results were previously reported in a separate paper for a larger parent sample (Knatz Peck et al., 2021).

Young adults completed the following measures at pre‐ and post‐ treatment: the EDE‐Q (Berg et al., 2012), the CIA (Bohn & Fairburn, 2008), and the McMaster FAD (Epstein et al., 2007). Exploratory analyses included data from the EDE‐Q, the CIA and the State Trait Anxiety Inventory (Spielberger et al., 1983) at pre‐treatment and 12‐month follow‐up. Cronbach's alphas for all measures were strong (alpha = 0.76–0.95) indicating good internal consistency.

### 2.3 Treatment description

YA‐TBT‐S is an intensive, multi‐family treatment specifically designed for YA and their parent(s). Parents and YA receive outpatient treatment (35 treatment hours delivered in five days), in a group format including multiple parents and YA. Treatment is developmentally adapted via parental involvement, YA focused life skills, and by narrow inclusion of YA only participants encouraging YA focused themes in group discussions. The treatment introduces a model of parent involvement that is tailored to developmental stage and addresses eating behaviour from a framework of temperament and neurobiology. Parents attend the entirety of treatment and receive YA‐focused parent skills training, neurobiology psychoeducation and activities, experiential activities focused on building effective YA‐parent relationships, dietary coaching (alongside YA), and in‐vivo, therapist‐assisted coaching during 21 therapeutic meals and snacks to ensure effective support (Hill et al., 2022; Knatz Peck et al., 2021; Wierenga et al., 2018).

### 2.4 Data analysis

Two‐tailed independent *t*‐tests and chi‐square tests were used to examine patterns of missing data from pre‐treatment to post‐treatment. Bonferroni corrections were applied to adjust for multiple comparisons; *p*‐values less than 0.006 were considered statistically significant for *t*‐tests, and *p*‐values less than 0.008 were considered statistically significant for chi‐square tests.

Descriptive statistics were used to characterise the sample of parent‐supports, calculating aggregated mean item scores (YA‐TBT‐S Acceptability) and summed scores of quantitative acceptability measures (CSQ).

***Primary analyses.*** Three 2 × 2 repeated measures ANOVAs were used to examine changes in the EDEQ/P‐EDEQ Global, P‐CIA/CIA, and FAD Family Functioning scales among parent‐supports and their children (i.e., the patients) from pre‐ to post‐treatment. Group (parent‐supports and patients) and time (pre‐treatment and post‐treatment) were the independent variables. Group differences across time points were examined with pairwise comparisons using a Bonferroni correction. Patients with avoidant restrictive food disorder diagnoses and their parent‐supports were excluded from the EDEQ/P‐EDEQ analysis due to the irrelevance of the measure to their symptoms. Pre to post‐treatment changes in the PVAS, a parent‐only measure, were examined with a two‐tailed paired sample *t*‐test. *p*‐values less than 0.05 were considered statistically significant. Partial eta squared and Cohen's d values were used to examine effect sizes.

***Exploratory analyses*.** Exploratory analyses were conducted to examine changes in parental self‐efficacy from pre‐ to post‐treatment as a predictor of eating disorder psychopathology, clinical impairment, and trait anxiety at 12‐month follow‐up using multiple linear regression models. Patient‐reported EDEQ global scale scores, STAI trait anxiety subscales scores (state and trait anxiety), and CIA total scores at 12‐month follow‐up were included as the dependent variable (reported as primary outcome variables in the primary study) (Knatz Peck et al., 2021). Parent‐reported change scores on the PVAS from pre‐ to post‐treatment were included as the independent variable in each model. Each model controlled for patient‐reported scores on the EDEQ, STAI, and CIA at pre‐treatment.

## 3 RESULTS

### 3.1 Missing data

Forty‐two supports (97.67%) completed the pre‐treatment questionnaire and 35 supports (81.40%) completed the post‐treatment questionnaire. Over three‐quarters of supports (78.57%, *n* = 33) completed both pre‐treatment and post‐treatment questionnaires. After adjusting for multiple comparisons, there were no differences in outcome scores or demographic characteristics between supports who completed the post‐treatment questionnaire and supports who did not.

### 3.2 Treatment acceptability

At post‐treatment, supports reported a mean CSQ score of 30.81/32 (*SD* = 1.62, range = 27–32), suggesting high acceptability of the treatment. Acceptability of the core tenets of the treatment are reported in Table 2 and suggest that parent‐supports found core treatment components to be highly acceptable.

**TABLE 2:** Parent YA‐TBT‐S acceptability questionnaire.

Treatment acceptability statements	Mean acceptability rating (SD)
I would recommend this treatment programme to others.	4.91/5 (0.30)
I would prefer additional group treatment sessions or exercises.	3.73/5 (0.98)
I would be willing to participate in additional group treatment sessions or exercises.	4.24/5 (0.75)
I enjoyed learning about the neurobiology of eating disorders through the group exercises.	4.75/5 (0.51)
The neurobiology exercises improved my understanding of my loved one's eating disorder.	4.61/5 (0.56)
I enjoyed activities in which I learnt and practiced effective communication with my loved one.	4.61 (0.56)
I feel equipped with new and better tools to support my loved one in their recovery.	4.67 (0.48)
I feel that I am able to better communicate with my loved one about his/her eating disorder.	4.61 (0.70)
I believe that developing the behaviour agreement with my loved one with help him/her be effective in recovery.	4.79 (0.55)
I am more confident in my ability to support my loved one throughout his/her recovery.	4.48 (0.71)
I feel that my role in my loved one's treatment has been clarified.	4.48 (0.67)
My relationship with my loved one has improved as a result of this treatment.	4.42 (0.75)
I believe my experience with this treatment will be helpful in decreasing the likelihood that my loved one will engage in eating disorder behaviours.	4.42 (0.56)
I enjoyed interacting with other patients and their supports during treatment.	4.79 (0.42)
I believe my experience with this treatment will be helpful in decreasing my loved one's anxiety and/or negative emotions and improving his/her ability to cope with these emotions.	4.27 (0.67)
I learnt skills and ideas from other clients and supports that I can now apply to my loved one's recovery.	4.55 (0.62)
I felt supported by the other group members.	4.70 (0.47)
I plan to continue my involvement in my loved one's recovery.	4.94 (0.24)

*Note:* Each item is rated on a 1–5 Likert scale, in which a higher score indicates greater acceptability.

### 3.3 Primary analyses: Parent‐reported changes in psychopathology

**FIGURE 1:**
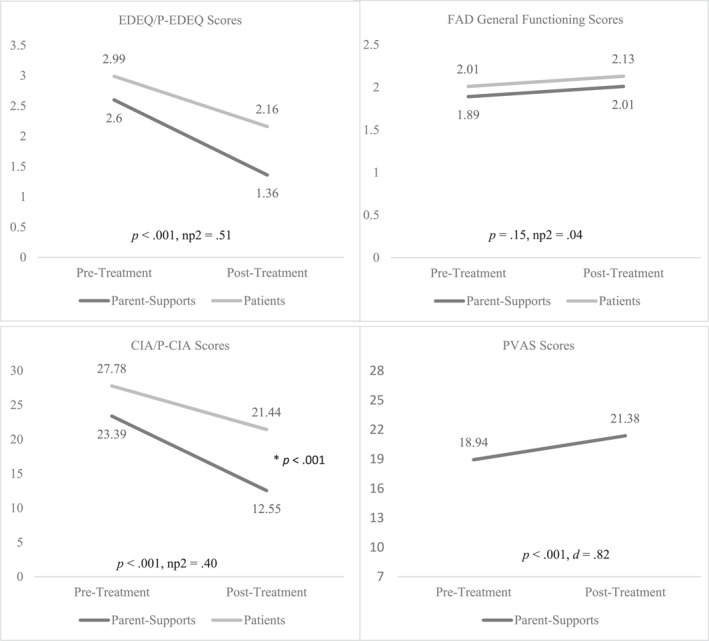
*p*‐values and effect sizes at the bottom of each graph represent the significance level and strength of the main effect of time. Asterisks designate significant between‐subjects effects where applicable. Measure abbreviations include: Eating Disorder Examination Questionnaire (EDE‐Q)/Parent‐report Eating Disorder Examination Questionnaire (EDEQ/PEDEQ), Family Assessment Device (FAD), Clinical Impairment Assessment (CIA)/Clinical Impairment Assessment‐ Parent report (CIA/CIA‐P), Parent Versus Anorexia (PVAS).

Data met parameters for parametric statistics. Results of RM ANOVA are displayed in Figure 1. Levene's Test of Equality indicated equal variances of eating disorder psychopathology, clinical impairment, and family functioning ratings at both pre‐treatment (*p*s > 0.05) and post‐treatment (*p*s > 0.05).

***Eating disorder psychopathology.*** Univariate tests revealed a significant main effect of time (*F*(1, 50) = 52.73, *p* < .001, $\eta_{p}^{2}$ = .51) but not group (*F*(1, 50) = 1.87, *p* = .18, $\eta_{p}^{2}$ = .04) on EDEQ scores with scores decreasing significantly from pre‐treatment to post‐treatment for both patient and parent reports. There was no significant time × group interaction effect (*F*(1, 50) = 2.10, *p* = .15, $\eta_{p}^{2}$ = .04), suggesting the relationship between time and EDEQ scores did not depend on group status.

***Clinical impairment.*** There was a significant main effect of time (*F*(1, 52) = 34.18, *p* < .001, $\eta_{p}^{2}$ = .40) and group (*F*(1, 52) = 4.22, *p* = .045, $\eta_{p}^{2}$ = .08) on CIA scores, suggesting clinical impairment scores decreased from pre‐ to post‐treatment but that scores differed by group. Pairwise comparisons showed that while there were no differences between patients and their parents on clinical impairment scores at pre‐treatment (*t*(54) = 1.13, *p* = .26), patients reported significantly more clinical impairment at post‐treatment (*t*(54) = 2.76, *p* = .26).

***Family functioning.*** There was no main effect of time (*F*(1, 52) = 2.10, *p* = 0.15, $\eta_{p}^{2}$ = 0.04) or group (*F*(1, 50) = 1.97, *p* = 0.17, $\eta_{p}^{2}$ = 0.04) on FAD general functioning scores nor was there a significant time × group interaction effect (*F*(1, 50) = 1.32, *p* = 0.26, $\eta_{p}^{2}$ = 0.03), suggesting family functioning did not change from pre‐ to post‐treatment for either patients or their parents. From pre‐ to post‐treatment, there was a significant parent‐reported increase in parental self‐efficacy (*t*(31) = −4.62, *p* < 0.001, *d* = 0.82). See Graph 1 for details of the above results. There were no differences in any of the aforementioned outcomes variables (at either pre‐treatment or post‐treatment) between supports who completed the treatment virtually and those who completed it in person (*p*s > 0.05).

### 3.4 Exploratory analyses

While all three examined regression models were significant (EDEQ: *R* ^2^ = 0.23, F(2,11) = 4.46, *p* = 0.04; STAI: *R* ^2^ = 0.31, F(2,17) = 3.84, *p* = 0.04; CIA: *R* ^2^ = 0.49, F(2,16) = 7.67, *p* = 0.005), after adjusting for patient‐reported scores at pre‐treatment, support‐reported changes in parental self‐efficacy from pre‐ to post‐treatment did not predict patient‐reported EDEQ global (*β* = −0.05, *p* = 0.82) trait anxiety (*β* = −0.12, *p* = 0.58), or clinical impairment (*β* = −0.30, *p* = 0.11) scores at 12‐month follow‐up.

## 4 DISCUSSION

This study examined parental acceptability of a brief, intensive, parent‐supported treatment programme for YA with EDs (YA‐TBT‐S) (Knatz Peck et al., 2021), and parental perception of change after participation using a naturalistic design. Results suggest that the treatment was highly acceptable to parents, with the substantial majority endorsing feeling more equipped to support their YA with recovery, improved self‐efficacy, and improvements in their YA‐child's ED psychopathology and impairment.

Our data suggest that parental involvement in YA care is feasible and appropriate, and that parents perceive a benefit to being involved. Improvements in self‐efficacy converge with findings from previous studies suggesting support person involvement in ED‐specific care improves parental self‐efficacy and caregiver skillfulness (Harrison et al., 2022; Sadeh‐Sharvit et al., 2018). Parents of YA often describe feeling helpless or uncertain on appropriate ways to support their newly appointed adult due to their legal status and progressive transition to greater autonomy. Improving parents' belief in their capacity to execute behaviours necessary to attaining recovery addresses this issue.

Both YA and parent reports of changes in YA ED‐related psychopathology and clinical impairment suggest a significant improvement in symptoms at treatment cessation, with no significant differences between parent report and YA report on ED psychopathology. However, parents reported significantly less clinical impairment than YA post‐treatment. This may be related to the lack of reliability and validity of parent report versions of these measures for YA, and/or a limitation in parents' capacity to accurately assess this phenomenology. On the other hand, it is possible that the parent report version provides incremental information, and/or information not captured by YA report due to under‐reporting (Drury et al., 2023; Schoen et al., 2012). Parent reports of impairment should be interpreted with caution and may be inflated or inaccurate, as they have not been validated for YA‐ED populations. Furthermore, no changes in perceptions of family functioning were observed from pre‐ to post‐treatment among patients or their parent‐supports, despite reporting clinically elevated level of problems (≥2).

Strengths of this study include evaluation of treatment from a novel perspective from a recovery‐adjacent support person, and preliminary evidence that a parent‐supported model of treatment for YA ED is acceptable for parents. This is one of very few studies to examine parental self‐efficacy in a sample of parents of YA (Dimitropoulos et al., 2018), and our results suggest that parental self‐efficacy was in fact improved. However, it is unclear whether these improvements impact change in psychopathology for YA.

Limitations of this study include its naturalistic design, the small sample size, a self‐referred sample likely resulting in a selection bias, and the fact that all outcomes are self‐reported by parents. Parent reported outcomes are also limited in their validity and reliability, as evidenced by the reported differences in parent and YA report of clinical impairment. Additionally, the treatment was brief in nature, and it is unclear whether parental report of changes would be durable over a longer follow‐up period. The changes reported at post‐treatment classify perceptions of change over the 5‐day treatment course. It is possible that these changes represent artificial treatment effects that may not be sustained after discharge. We did not evaluate parental perceptions of change at 12‐month follow up because the majority of participants transitioned to other psychological treatments at discharge, which is a significant confound in the reports of improvement.

Future studies should include additional parental measures to assess improvements in functioning and distress and relationships with outcomes. Randomized, controlled evaluations of parent‐supported treatment versus individual treatment with an adequately powered sample are needed to determine whether the addition of parents to treatment in a developmentally aligned manner can enhance outcomes, improve dropout and utilization, and reduce the risk of relapse.

In conclusion, results provide preliminary evidence that parents of YA with ED perceive treatment to improve their ability to support ED recovery, and perceived improvement in ED‐related symptoms and behaviours during treatment aligned with YA reports.

## CONFLICT OF INTEREST STATEMENT

The authors have no conflict of interests to report.

## Data Availability

Data available upon request.
